# “Hair in the Bladder”: An Unusual Finding

**DOI:** 10.1089/cren.2017.0012

**Published:** 2017-03-01

**Authors:** Luca Cindolo, Maida Bada, Roberto Bellocci, Piergustavo De Francesco, Pietro Castellan, Francesco Berardinelli, Fabio Neri, Luigi Schips

**Affiliations:** ^1^ Department of Urology, ASL 2 Abruzzo, Chieti, Italy.; ^2^ Department of Surgical Pathology, “S.Pio da Pietrelcina” Hospital, Vasto, Italy.

**Keywords:** trichobezoar, bladder, trichotillomania, chronic indwelling catheter, intermittent catheterization, urinary tract infection

## Abstract

Trichobezoar is a rare condition whereby a hairball is found in the human stomach or gastrointestinal tract, most frequently in young women, mainly in association with a psychiatric disorder. Trichobezoar cases have also been reported in the bladder and represent a rare complication of foreign bodies, called “hair nidus or hair ball,” in patients with chronic catheter. Approximately 10% to 15% of patients on long-term urethral catheter or clean intermittent self-catheterization develop urinary tract stones. In a small minority of cases, bladder stones can develop around a foreign body that was introduced into the bladder. In the literature, there are few cases of foreign bladder bodies that formed stones over a hair nidus. Recognizing this condition can optimize the patient's quality of life. Herein, we present a case of a 71-year-old Caucasian male with a long-term catheter in hypocontractile urinary bladder secondary to injury of pelvic plexus after rectal surgery. He had a bladder stone caused by hair encrusted together. Hair is introduced into the bladder either by adherence to the catheter directly or by overlying the urethral meatus and being pushed internally. Regular hygiene and shaving of pubic area represent effective preventive measures to reduce this kind of complications in patients with chronic indwelling catheter or under a self-catheterization regimen.

## Case Presentation

### Clinical history

A 71-year-old Caucasian man, affected by mild hypertension and taking beta blockers and low-dose aspirin for primary prevention, was referred to our clinic for recurrent urinary tract infections (UTIs) with severe dysuria and bladder pain/discomfort.

After a Miles' operation for rectal cancer in 2008, the patient developed a hypocontractile bladder. An initial period of clean intermittent catheterization was followed by 18F Foley indwelling catheter for 7 years.

A specialized nurse changed the catheter monthly. The physical examination was substantially normal, systemic parameters within normal limits, the catheter was *in situ*, and the digital rectal examination was impossible because of the closed anus. The colostomy was well functioning and trophic. The abdominal scar was smooth and dry. No other thoracic or abdominal abnormalities were obtained during inspection, auscultation, percussion, or palpation. Ultrasound showed a normal upper urinary tract with reduced capacity bladder with a 2 cm bladder stone and a small volume prostate (estimated volume: 28 cc) with calcifications. Preliminary analyses were normal with the exception of the urine culture that was positive for *Enterobacter cloacae* (>10^6^ CFU/mL). The serum prostate specific antigen level was 1.1 ng/mL.

**FIG. 1.**
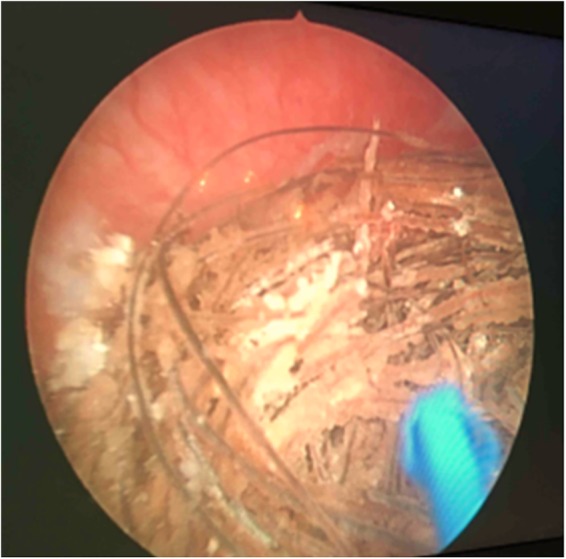
Endoscopic view of the “hair ball” before lasertripsy.

**FIG. 2.**
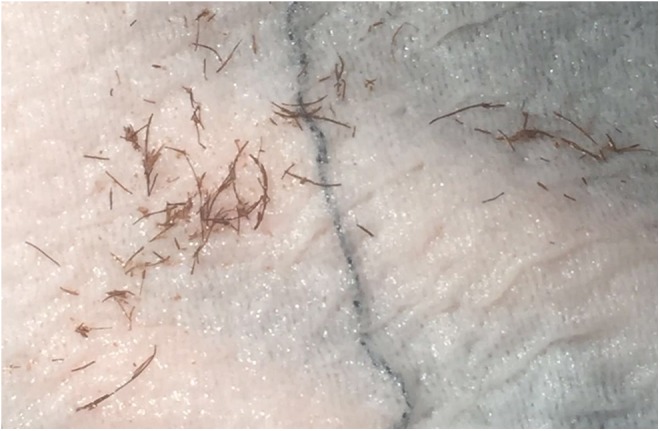
Extracted fragments after lasertripsy.

**FIG. 3.**
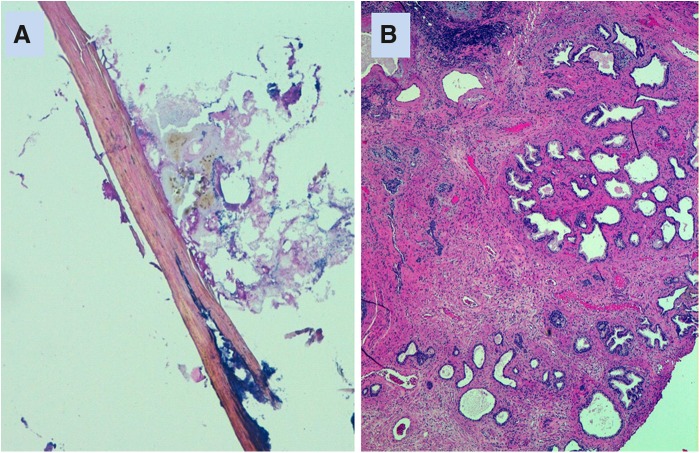
**(A)** Microscopic appearance of hair shaft with amorphous material (HE, 10 × ). **(B)** Benign prostatic hyperplasia and atrophic prostatic ducts with surrounding sclerosis and chronic inflammation (HE, 4 × ). HE, hematoxylin and eosin.

A cistolithotripsy with an endoscopic resection of the small prostate was planned and done in July 2016. A holmium lasertripsy was easily performed followed by a standard monopolar resection of the prostate. Endoscopy confirmed the hair nidus in the bladder (Fig. 1). Gross examination of the evacuated fragments confirmed the hypothesis of a trichobezoar in the bladder and the sample was sent for microscopic evaluation (Figs. 2 and 3A). The postoperative period was characterized by 2 days of fever (max 38.8°C) that was treated with antibiotics (sterile urine culture), and the patient was discharged on the fourth postoperative day in good general conditions, without catheter, with a mild urinary incontinence. The histopathology examination confirmed a benign prostatic hyperplasia with chronic inflammation (Fig. 3B). The patient was seen in our office 10 days after the treatment, and then at 1 and 6 months. At the last follow-up, the patient was free from the catheter, he reported a good subjective satisfaction with an improvement of incontinence (maximum 1 pad/day). Urine culture was negative and uroflow was normal, without postvoid residual.

## Discussion

In patients with spinal cord injury or in case of pelvic plexus damage, intermittent self-catheterization or permanent catheter is often required for emptying the bladder and preventing complications such as urine retention, renal function impairment, and calculi formation. Bladder stone is one of the common complications with an estimated incidence ranging between 10% and 15%, but bladder stone on foreign bodies (misplaced silk suture, eroded surgical mesh, fragments of clothing, intrauterine devices, ureteral stent, and patients with Foley catheters) have a reported incidence of <0.8%.^1,2^ The stone composition is usually calcium phosphate.^1,2^

One of the more recent articles in the literature of a hairball with a secondary calculus was reported in 2004^3^: the patient was a 30-year-old woman with neurogenic bladder secondary to spina bifida, necessitating long-term catheter drainage, which lead to *Escherichia coli* infection in urine and a “hairball” stone in the bladder. Joshi reported three cases of bladder stones in patients with spinal cord injury in intermittent catheterization. The authors explained how the pubic hair can be introduced into the bladder either by adhering directly to the lubricated catheter or by overlying the urethral meatus and being pushed into the bladder.^4^ Impaired tactile discrimination in these patients can also make the sensation of a small hair less noticeable as compared with persons with normal sensation.^4^ Acting as a foreign material, hair in the bladder is an ideal locus for crystalline precipitation, which may then be perpetuated by the reaction of the urothelium to the extraneous material.^1–4^

In general, an inappropriate technique like improper position in bed or wheelchair during intermittent catheterization, poor visibility of the urethral meatus and glans, the presence of Foley catheter, limited fluid intake, concentrated urine, and a higher frequency of UTI may be considered as risk factors for stone formation around foreign bodies.^4,5^ In our case, the patient had a history of indwelling catheter for 7 years with recurrent UTI and he was assisted by a specialized nurse. As already described, the introduction of hair worked as a locus to initiate stone formation. The presence of bacteria in a hypersaturating urine probably facilitated the stone's growth.

The important message from this case is to emphasize the role of nurses, physicians, and urologists to instruct patients and caregivers on proper hygiene during self-catheterization or routine change of chronic catheter to prevent complications. As suggested by Joshi and Mittal, an annual urologic surveillance seems to adequately prevent and treat any kind of urologic long-term complications.^4^ Of course, renal function tests, urinary tract ultrasound, and also cystoscopy screening are recommended in high-suspicious cases.^4^ Moreover, as suggested by Perz and colleagues and previously demonstrated by Amendola and colleagues, removal of pubic hairs either by regular shaving, diathermy destruction, or laser depilation has been suggested to prevent this complication from recurring.^2,5^ Although shaving of pubic hair may not be acceptable to some patients, we should discuss this topic with the patients and their caregivers.

## Conclusion

In this report, we present a case of “hair ball” in the bladder endoscopically managed by laser lithotripsy. As the preoperative imaging techniques are poorly diagnostic, a periodic cystoscopy should be suggested in cases of chronic self-catheterization or indwelling catheter in highly suspicious cases. To prevent this kind of complication, patient education and nurse assistance represent the most important solution.

## Abbreviations Used

HE: hematoxylin and eosin
UTI: urinary tract infection
